# Editorial: Interplay between glucose and lipid homeostasis in metabolic health

**DOI:** 10.3389/fendo.2026.1890843

**Published:** 2026-06-16

**Authors:** Wenbo Bian, Qiaoran Liu, Zhenyu Yao

**Affiliations:** 1 Key Laboratory of Endocrine Glucose & Lipids Metabolism and Brain Aging, Ministry of Education, Jinan, Shandong, China; 2 Department of Endocrinology, Shandong Provincial Hospital Affiliated to Shandong First Medical University, Jinan, Shandong, China; 3 Shandong Clinical Research Center of Diabetes and Metabolic Diseases, Jinan, Shandong, China; 4 Department of General Surgery, The First Affiliated Hospital of Shandong First Medical University & Shandong Provincial Qianfoshan Hospital, Jinan, Shandong, China; 5 Key Laboratory of Metabolism and Gastrointestinal Tumor, the First Affiliated Hospital of Shandong First Medical University, Jinan, Shandong, China

**Keywords:** metabolic flexibility, glucose lipid homeostasis, metabolic diseases, diet and exercise interventions, composite metabolic indices

Metabolic flexibility is a core feature of metabolic health, referring to the capacity to switch efficiently between glucose and lipid substrates in response to nutritional intake, hormonal status, and energetic demand (1). In healthy states, the body preferentially utilizes glucose and stores energy after feeding, while enhancing fatty acid mobilization and oxidation during fasting or exercise. When this flexibility is impaired, nutrient excess may be redirected toward insulin resistance, ectopic lipid deposition, mitochondrial dysfunction, and chronic inflammation. Thus, the interplay between glucose and lipid homeostasis provides an important framework for understanding obesity, diabetes, steatotic liver disease, and other metabolism-related disorders.

Public databases, large cohorts, and multi-omics resources have increasingly enabled metabolic risk to be assessed as an integrated phenotype rather than through isolated biomarkers. These developments have facilitated the use of accessible composite indices, including the triglyceride-glucose index (TyG), visceral adiposity index (VAI), lipid accumulation product (LAP) and metabolic score for insulin resistance (METS-IR), to characterize integrated risks arising from glucose-lipid abnormalities (2, 3). Rather than replacing established diagnostic tools, these indices may complement existing approaches for metabolic risk stratification in large populations.

Several articles in this Research Topic reflect this trend. In steatotic liver disease, Ma et al. found that TyG-derived anthropometric composite indices, particularly the TyG-waist-to-height ratio, were associated with high-risk non-alcoholic steatohepatitis (NASH). Duan et al. suggested that remnant cholesterol and TyG were associated with non-alcoholic fatty liver disease (NAFLD) severity and fibrosis-related indices at different levels. These two studies are consistent with the view that steatotic liver disease is not merely hepatic lipid deposition, but may represent an important hepatic manifestation of systemic glucose-lipid dysregulation. Its risk assessment may therefore need to move beyond single markers toward an integrated evaluation of metabolic burden, fat distribution, and tissue injury. The transition from NAFLD/NASH to MASLD/MASH also reflects a shift from viewing steatotic liver disease as a localized hepatic disorder toward understanding it as part of systemic metabolic dysfunction (4). Beyond steatotic liver disease, the prognostic relevance of glucose-lipid composite indices was also explored in acute decompensated heart failure. He et al. and Jian et al. examined the relationships of TyG/HDL-C and FPG/HDL-C ratios with short-term mortality, respectively, suggesting that these indices may provide additional information for risk stratification in high-risk cardiovascular settings.

Diet provides a physiological context in which metabolic flexibility is repeatedly challenged by nutrient quantity, quality, and timing. Comparative studies of low-fat and low-carbohydrate diets suggest that macronutrient composition alone may be insufficient to predict individual weight and metabolic responses (5). These observations have redirected attention toward dietary quality, fatty acid composition, carbohydrate quality, meal timing, and individualized metabolic responses. Within this framework, dietary patterns and meal-timing interventions help clarify how diet may influence metabolic flexibility. The Mediterranean diet is one of the most extensively studied dietary patterns, with randomized and meta-analytic evidence suggesting potential benefits for type 2 diabetes prevention and metabolic syndrome components, including adiposity, lipid levels, glucose metabolism, and blood pressure (6). Alternate-day fasting and 16:8 time-restricted eating has also attracted attention, but their additional benefits, long-term adherence, and suitable populations remain to be clarified (7, 8).

Two diet-related studies in this Research Topic complement this perspective. Liu et al. showed that postprandial free fatty acid and cortisol responses differed by macronutrient composition, suggesting that dietary fat exposure may participate in metabolic regulation through endocrine pathways. Sun et al. further showed in an animal study that, under high-energy dietary conditions, a higher fat proportion aggravated obesity, insulin resistance, dyslipidemia, and type 2 diabetes, with mechanisms involving hepatic mitochondrial dysfunction and lipid metabolic reprogramming. These findings suggest that diet-related metabolic risk depends not only on nutrient quantity, but also on substrate handling capacity, endocrine adaptation, and tissue-specific metabolic responses.

Exercise is a key setting for studying and improving metabolic flexibility. Beyond increasing energy expenditure, regular exercise improves the ability to switch between glucose and lipid substrates. During exercise, skeletal muscle dynamically recruits blood glucose, muscle glycogen, circulating free fatty acids, and intramyocellular lipids. Previous studies suggest that this substrate-switching capacity is closely related to insulin sensitivity, adiposity, and physical fitness (1). More recently, exercise metabolism research has moved from single biomarkers toward cross-tissue and multi-omics analyses, revealing systemic molecular adaptations induced by exercise.

Exercise may improve glucose-lipid homeostasis by coordinating lipid storage, mobilization, and oxidation rather than simply reducing lipid accumulation. The “athlete’s paradox” shows that endurance-trained individuals may maintain favorable insulin sensitivity despite relatively high intramyocellular lipid content, suggesting that lipid droplet size, localization, turnover, and coupling with mitochondria may be more important than total lipid quantity itself (9). This concept is consistent with the review on PLIN-mediated lipid droplet-mitochondria interactions by Liu et al. in this Research Topic. Exercise-induced lactate is also not merely an energy substrate, but may act as an exerkine involved in inter-tissue communication and influence brain function and neuroplasticity, as discussed by Zhu et al. Thus, exercise can be understood as a systemic metabolic stimulus connecting skeletal muscle, liver, adipose tissue, the cardiovascular system, and brain function.

Other contributions extended this framework to acute pancreatitis and polycystic ovary syndrome. Fang et al. and Du et al. broadened the clinical scope of glucose-lipid homeostatic imbalance from the perspectives of inflammatory injury and reproductive endocrine dysfunction, respectively. Given differences in study design and evidence level, these findings should be regarded as complementary clues rather than definitive conclusions.

In summary, this Research Topic illustrates the complexity of glucose-lipid homeostatic interactions across disease and intervention contexts. Beyond its descriptive role, metabolic flexibility may provide an organizing framework linking composite metabolic indices, dietary exposures, exercise adaptations, and organ-specific disease phenotypes. Future research should integrate public databases, longitudinal cohorts, multi-omics technologies, mechanistic experiments, and intervention trials to validate emerging composite indices and clarify the long-term effects of dietary, exercise, and integrated lifestyle interventions. Identifying individualized risk profiles and modifiable metabolic windows may provide a more reliable basis for precision prevention and treatment of metabolic diseases, and may ultimately contribute to greater metabolic resilience.
